# Alignment of United States Emergency Medicine Programs’ Pregnancy, Parental Leave, and Lactation Policies With Recent Consensus Recommendations

**DOI:** 10.1016/j.acepjo.2026.100428

**Published:** 2026-06-13

**Authors:** Laura G. Shepherd, Michelle D. Lall, Shivam Champaneria, Justin Davis, Michael Dinh, Carney Flinn, Kristen Helmsdoerfer, Rasim Kazic, Kellsa Mbah, Alycia N. Montgomery, Seyjil Morrow, Jeffery Peters, Ryan Shepherd, Rebecca Sobolewski, Michael Ysit, William Manning, Jin H. Han

**Affiliations:** 1 Department of Emergency Medicine, Vanderbilt University Medical Center, Nashville, Tennessee, USA; 2 Department of Emergency Medicine, Emory University School of Medicine, Atlanta, Georgia, USA; 3 Geriatric Research, Education, and Clinical Center and the Veterans’ Wellbeing through Innovation, Systems Science and Experience in Learning Health Systems, Tennessee Valley Healthcare Center, Nashville, Tennessee, USA

**Keywords:** pregnancy, parental leave, lactation, parental policies

## Abstract

**Objectives:**

Many emergency physicians start families. Consensus recommendations to support physician pregnancy, parental leave, and lactation for emergency medicine (EM) programs were recently developed. This study aimed to determine how well EM programs’ existing parental policies align with these recommendations for resident and attending physicians across US emergency departments.

**Methods:**

A 53-item REDCap survey evaluated policies for pregnancy, parental leave, and lactation in university- and community-based EM programs with residencies. The survey was emailed to faculty chairs and residency program directors at the 282 programs listed in the Fellowship and Residency Electronic Interactive Database Access database, with at least 4 contact attempts (one by phone) from September 2024 to February 2025.

**Results:**

A total of 102 (36%) residency program surveys and 83 (29%) attending group surveys were completed. Most respondents limited overnight shifts in the third trimester for resident and attending physicians (69% and 76%), though fewer, did so in the first trimester (39% and 34%). In the postnatal period, nonbirthing parents received compensated leave in 68% and 57% of resident and attending programs, respectively. Access to compensated leave for adoptive parents and parents by surrogate ranged from 43% to 52% for resident programs and 28% to 42% for attending programs, respectively. Most programs had written lactation policies supporting breastfeeding indefinitely, though access to adequate lactation space and the ability to step away from patient care varied. A minority covered assisted reproductive therapies.

**Conclusion:**

EM programs’ existing parental policies do not consistently align with recently published consensus-driven parental policy recommendations. More work is needed to increase the adoption of recommendations and understand the impact of adherence on parental and fetal outcomes.

**The Bottom Line**

In a national review of academic and community emergency departments with residency programs, we evaluated current policies for physician pregnancy accommodations, parental leave, adoption and surrogacy, and lactation support. Policies varied widely and often did not align with recently published consensus recommendations. These findings indicate gaps in institutional support during pregnancy and early parenthood and underscore the need for the implementation of comprehensive, transparent parental policies for emergency medicine physicians.

## 1 Introduction

### 1.1 Background

With many physicians and physician trainees navigating parenthood alongside patient care, the need for comprehensive, data-driven policies to support physician parents has never been greater. Medical training often spans beyond a decade, overlapping with peak childbearing years.^1^ The traditional notion of delaying parenthood during residency and as an early attending is being challenged by increasing representation of female physicians and gender equity advocacy.^2^^,^^3^ There is a growing awareness of the risks associated with postponing childbearing until after training with the number of resident physicians planning on starting a family during training at an all-time high.^1^^,^^4^

### 1.2 Importance

Substantial and mounting evidence highlights the elevated risk of adverse maternal and fetal health outcomes for pregnant physicians and physician trainees facing extended work hours, overnight shifts, and limited parental leave. These occupational factors have been associated with increased rates of miscarriage, hypertensive disorders of pregnancy, placental abruption, fetal growth restriction, low birth weight, preterm delivery, sleep deprivation, depression, anxiety, and decreased rates of breastfeeding.^5^, ^6^, ^7^, ^8^, ^9^, ^10^, ^11^, ^12^, ^13^, ^14^

As a physician shortage crisis looms, safeguarding the well-being of physicians and their children is both a moral imperative and a top priority for the functioning of our health care system.^15^ Emergency physicians are leaving clinical medicine for myriad reasons amid an increasingly difficult work environment. The unique challenges facing physician parents contribute to this attrition, with parent physicians reporting increased stress and decreased career satisfaction after childbirth.^16^^,^^17^ There is growing recognition of the critical importance of family-inclusive policies on physician well-being, retention, and physical health, and forward momentum for the development of such policies and practices, where occupational exposures, erratic schedules, and high-stress interactions are daily occurrences.^18^, ^19^, ^20^, ^21^, ^22^, ^23^

Despite evidence of substantial physical and psychological health benefits from supportive policies and paid parental leave, comprehensive parental policies addressing pregnancy accommodations, parental leave, parenthood by adoption or surrogacy, and lactation are lacking across medicine.^7^^,^^24^^,^^25^ In 2024, consensus recommendations for pregnancy, parental leave, adoption, surrogacy, and lactation policies for emergency physicians were put forth by a working group representing the American College of Emergency Physicians (ACEP) Well-Being Committee and the Society for Academic Emergency Medicine Academy for Women in Academic Emergency Medicine.^26^ However, the extent to which academic and community emergency department's (ED's) existing parental policies for resident physicians and attendings align with these recommendations is unknown.

### 1.3 Goals of This Investigation

Our objective was to evaluate the current state of parental policies for residents and attending physicians across EDs with residency programs to understand how they align with recently published consensus recommendations.

## 2 Methods

### 2.1 Study Design

This was a survey study conducted from September 13, 2024, to February 28, 2025. We included university- and community-based EDs in the United States (US) that had an Accreditation Council for Graduate Medical Education accredited emergency medicine (EM) residency program listed in Fellowship and Residency Electronic Interactive Database Access (FREIDA) provided by the American Medical Association. Programs were excluded if accurate contact information could not be obtained. This study was approved as exempt by our institutional review board review.

### 2.2 Survey

A structured survey was developed based on “Consensus-Driven Recommendations to Support Physician Pregnancy, Adoption, Surrogacy, Parental Leave, and Lactation in Emergency Medicine.”^26^ Initially, each of the 17 recommendations was adapted into ≥1 corresponding questions by several members of the study team. The senior investigators then reviewed all survey questions to ensure clarity, accuracy, and alignment with the parental policies. During this phase, questions were refined to balance thoroughness with feasibility and the goal of limiting survey completion time to <20 minutes. The survey was then pilot-tested by 6 resident and attending physicians, and iterative refinements to the survey were made based on their feedback.

The survey included questions about work modifications during the prenatal period; parental leave; lactation; policies around adoption, surrogacy, and assisted reproductive technologies; and return-to-work accommodations. Specifically, questions about work modifications during the prenatal period included: excused absences and support for medical appointments for residents, limitations to maximum weekly clinical hours and overnight shifts, exemptions from mandatory work above contracted hours, and departmental practices for directing care of patients with possible tuberculosis, meningitis, or herpes zoster toward nonpregnant physicians.

Questions regarding parental leave after birth, adoption, or surrogacy addressed the number of weeks off and the presence of compensated leave for birthing and nonbirthing parents. Questions related to lactation included the existence of a written lactation policy, the ability to step away from clinical duties for milk expression, whether minimal or no single-coverage shifts were scheduled while the physician parent was lactating, the duration of lactation accommodations, and access to a hospital-grade breast pump and a private lactation space equipped with a phone and/or computer for work. Questions addressing return-to-work accommodations after the birthing parent’s medical leave included scheduling accommodations for overnight shifts, overtime or jeopardy call, and total shift number, as well as any modifications to relative value unit expectations or bonus compensation. Additional questions evaluated the inclusion of fertility treatment with assisted reproductive technology within the benefits package.

### 2.3 Data Collection

The surveys were administered by the Emergency Medicine Post Graduate Year 1 (PGY-1) class as part of the Vanderbilt University Medical Center Research Immersion Program.^27^ Email links to electronic surveys were first sent to program directors, coordinators, chairpersons, or medical directors for residency programs and attending groups, respectively, via Research Electronic Data Capture (REDCap). After the initial email, 2 additional follow-up emails were sent. If there was no response, the research team attempted to complete the survey by phone.

The FREIDA database only provides contact information for program directors and coordinators of residency programs. To obtain parental policies data for attending physicians, the intern class searched these programs’ departmental or hospital websites for chairperson or medical directors’ contact information. If no contact information was available on the website, then an email was sent to the respective program director or coordinator to obtain this contact information.

### 2.4 Statistical Analysis

This study is descriptive in nature. No statistical comparisons were performed. Measures of central tendency and dispersion were reported as medians and interquartile ranges (IQR). Frequency and proportions were reported for categorical variables. All analyses were performed using SAS Version 9.4 (SAS Institute).

## 3 Results

A total of 282 US EM programs with residencies were in the FREIDA database (Fig) and contact information for residents and attendings was found for 282 and 278 programs, respectively. Of the programs surveyed, 102 programs (36%) completed the survey for resident policies, and 83 programs (29%) completed the survey for attending policies. Characteristics for programs that completed the resident and attending parental policy surveys can be seen in Table 1. Programs that did not complete the surveys were less likely to be university-based programs and more likely to be community-based programs without a university affiliation (Table S1).

**Figure:**
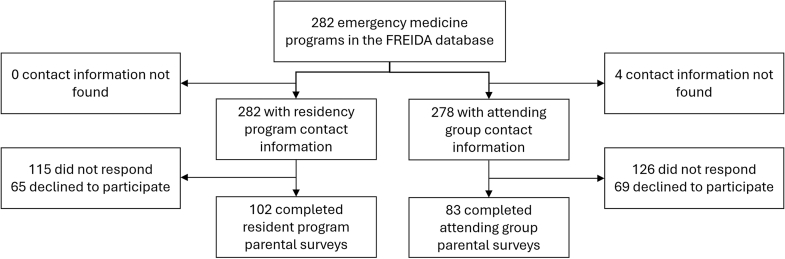
Enrollment flow diagram.

**Table 1:** Characteristics of survey participants.

Program characteristics	Residency programs n = 102	Attending groups n = 83
Type of program, n (%)		
University	50 (49.0)	41 (49.4)
Community with university affiliation	35 (34.3)	35 (42.2)
Community without university affiliation	17 (16.7)	7 (8.4)
Missing/unknown	0 (0.0)	0 (0.0)
Survey respondent, n (%)		
Residency program director	94 (92.2)	0 (0.0)
Assistant program director	1 (1.0)	0 (0.0)
Residency program manager	5 (4.9)	0 (0.0)
Chief/administrative resident	1 (1.0)	0 (0.0)
Chair	0 (0.0)	56 (67.5)
Vice-chair	0 (0.0)	8 (9.5)
Medical director	0 (0.0)	6 (7.2)
Other	1 (1.0)	13 (15.7)
Residency length, n (%)		
3-years	80 (78.4)	N/A
4-years	22 (21.6)	N/A
No. of residents, median (IQR)	36 (27, 48)	36 (30, 48)
No. of faculty, median (IQR)	43 (20, 65)	40 (24, 56)

The full questionnaire and its responses on prenatal accommodations, parental leave, lactation support, return-to-work practices, and fertility benefits are shown in Tables S2-S5. Overall, the results demonstrate variability in support for parenthood across EM. Highlights of the survey responses are summarized below.

### 3.1 Work Modifications During the Prenatal Period

Support for work accommodations during pregnancy varied by trimester and professional role (Table 2). During the first trimester, 40 (39.2%) residency programs and 28 (33.7%) attending groups allowed pregnant individuals to opt out of overnight shifts. More accommodations were offered in the third trimester, with 70 (68.6%) residency programs and 62 (74.7%) attending groups exempting pregnant physicians from overnight shifts.

**Table 2:** Prenatal work modifications for residents and attendings in emergency medicine programs in the United States.

Prenatal work modifications	Residents	Attendings
Overnight shifts are limited in the first trimester, n (%)		
No	58 (56.9)	49 (59.0)
Yes	40 (39.2)	28 (33.7)
Unknown	4 (3.9)	6 (7.2)
Overnight shifts are limited in the third trimester, n (%)		
No	23 (22.6)	15 (18.3)
Yes	70 (68.6)	62 (75.6)
Unknown	9 (8.8)	5 (6.1)
Clinical hours per week are limited in the third trimester, n (%)		
No	78 (76.5)	72 (87.8)
Yes	17 (16.7)	8 (9.8)
Unknown	7 (6.9)	2 (2.4)
Of those with limitations on clinical hours (n = 17 for residents and n = 8 for attendings), maximum number of clinical hours per week, median (IQR)	60 (57, 60)	60 (32, 60)
Pregnant persons in the third trimester are exempt from mandatory overtime or jeopardy/back-up call, n (%)		
No	44 (43.1)	28 (34.2)
Yes	38 (37.3)	45 (54.9)
Unknown	20 (19.6)	9 (11.0)
For pregnant persons (all trimesters), the frequency of patients with possible tuberculosis, meningitis, or herpes zoster directed toward nonpregnant health care physicians, n (%)		
Never	6 (5.9)	9 (11.0)
Always	50 (49.0)	22 (26.8)
Sometimes	19 (18.6)	28 (34.2)
Unknown	27 (26.5)	23 (28.1)
For residents, medical appointments are excused absences, n (%)		
No	23 (22.6)	-
Yes	75 (73.5)	-
Unknown	4 (3.9)	-

During the third trimester, 17 (16.7%) residency programs and 8 (9.8%) attending groups had limitations on the number of clinical hours worked per week. Of those with limitations, the median (IQR) maximum hours per week were 60 (57, 60) and 60 (32, 60) clinical hours per week, respectively. Exemptions from mandatory overtime or back-up call during the third trimester were reported in 38 (37.3%) residency programs and 45 (54.9%) attending groups. Medical appointments were considered excused absences for 75 (73.5%) residency programs. Patients with possible tuberculosis, meningitis, or herpes zoster were *always* or sometimes directed toward nonpregnant health care physicians in 69 (67.6%) residency programs and 50 (61.0%) attending groups.

### 3.2 Postnatal Parental Leave

Paid parental leave policies varied across institutions and between birthing and nonbirthing parents in the postnatal period (Table 3). Resident physicians who were birthing parents received a median (IQR) of 6 (6, 8) weeks of paid leave. Attending physicians who were birthing parents received a median (IQR) of 8 (6, 12) weeks. Paid leave was more limited for nonbirthing parents. For residents, the nonbirthing parent received compensated leave in 69 (67.7%) programs with a median (IQR) of 6 (4, 6) weeks of leave provided. For attending groups, the nonbirthing parent received compensated leave in 47 (56.6%) groups, with a median (IQR) of 8 (6, 12) weeks provided. The percentage of respondents who provided paid leave for adoptive parents and parents by surrogate ranged from 43.1% to 52.0% for resident programs and 27.7% to 42.2% for attending groups.

**Table 3:** Postnatal parental leave for residents and attendings in emergency medicine programs in the United States.

Postnatal parental leave policies	Residents	Attendings
Postnatal parental leave		
Weeks of paid leave for birthing parent, median (IQR)	6 (6, 8)	8 (6, 12)
Nonbirthing parents with paid leave, n (%)		
Nonbirthing parent	69 (67.6)	47 (57.3)
Adoptive parent #1	53 (52.0)	35 (42.7)
Adoptive parent #2	49 (48.0)	27 (33.7)
Parent #1 by surrogacy	47 (46.1)	28 (34.1)
Parent #2 by surrogacy	44 (43.1)	23 (28.0)
Among those with paid leave, weeks of paid leave provided for nonbirthing parents, median (IQR)^a^		
Nonbirthing parent (n = 66 for residents, n = 44 for attendings)	6 (4, 6)	8 (6, 12)
Adoptive parent #1 (n = 49 for residents, n = 33 for attendings)	6 (6, 7)	12 (6, 12)
Adoptive parent #2 (n = 46 for residents, n = 25 for attendings)	6 (6, 7)	12 (6, 12)
Parent #1 by surrogacy (n = 43 for residents, n = 28 for attendings)	6 (6, 8)	8 (6, 12)
Parent #2 by surrogacy (n = 41 for residents, n = 20 for attendings)	6 (6, 8)	8 (6, 12)

a Not all respondents knew the amount of paid leave provided.

### 3.3 Lactation

Most resident programs (78.2%) and attending groups (63.4%) reported a written lactation policy, with the majority extending indefinitely without a limit on the duration of accommodations (84.8% of resident programs, 69.2% of attending groups) (Table 4). Almost all programs provided a dedicated lactation space near or within the department (96.1% of resident programs, 89.0% of attending groups). However, the accommodations provided within these spaces varied. Among those that had a dedicated lactation space, a refrigerator for breast milk storage was available at 88 (89.8%) residency programs and 54 (74.0%) attending groups. Additionally, 65 (66.3%) residency programs and 39 (53.4%) attending groups reported that lactation rooms were equipped with a desk, telephone, and computer linked to the electronic health record, enabling continued clinical documentation during lactation breaks.

**Table 4:** Lactation policies for residents and attendings in emergency medicine programs in the United States.

Lactation policies	Residents	Attendings
Written lactation policy, n (%)		
No	9 (8.9)	16 (19.5)
Yes	79 (78.2)	52 (63.4)
Unknown	13 (12.9)	14 (17.1)
Duration of accommodations for lactating parents, n (%)		
Limited	6 (7.6)	6 (11.5)
Indefinite	67 (84.8)	36 (69.2)
Unknown	6 (7.6)	10 (19.2)
Dedicated private lactation space in or near ED, n (%)		
No	1 (1.0)	7 (8.5)
Yes	98 (96.1)	73 (89.0)
Unknown	3 (2.9)	2 (2.4)
Among programs with dedicated lactating space, accommodations that are provided (n = 98 for residents, n = 73 for attendings), n (%)		
Sink	63 (64.3)	42 (57.5)
Refrigerator	88 (89.8)	54 (74.0)
Comfortable chair	96 (98.0)	71 (97.3)
Closet space for pumping equipment	49 (50.0)	37 (50.7)
Desk with phone and computer	65 (66.3)	39 (53.4)
Lactating physicians can safely schedule breaks from clinical coverage for the expression of breast milk, n (%)		
No	6 (5.9)	4 (4.9)
Yes	91 (89.2)	71 (86.6)
Unknown	5 (4.9)	7 (8.5)
Programs that eliminate single-coverage shifts for lactating physicians, n (%)		
No	37 (36.3)	43 (52.4)
Yes	39 (38.2)	27 (32.9)
Unknown	26 (25.5)	12 (14.6)
Programs that minimize single-coverage shifts for lactating physicians, n (%)		
No	34 (33.3)	33 (40.2)
Yes	43 (42.2)	39 (47.6)
Unknown	25 (24.5)	10 (12.2)
Privacy screen for lactating physicians to pump in the charting area in emergency department, n (%)		
No	77 (75.5)	66 (80.5)
Yes	14 (13.7)	11 (13.4)
Unknown	11 (10.8)	5 (6.1)

ED, emergency department.

Fewer programs reported *eliminating* single-coverage shifts for lactating physicians for up to one year postpartum (38.2% of residency programs and 32.9% of attending groups). However, a larger percentage of programs reported *minimizing* single-coverage shifts for lactating physicians for up to one year postpartum (42.2% of residency programs and 47.6% of attending groups). Though few respondents reported having access to a privacy screen at the work area or charting space to allow continuation of work while expressing milk (13.7% for residency programs and 13.4% for attending groups), most (89.2% for resident programs and 86.6% for attending groups) reported being able to safely take breaks from clinical coverage for the expression of breastmilk.

### 3.4 Return-to-Work Accommodations

Postpartum return-to-work support was limited across both residency programs and attending groups (Table 5); 48 (47.1%) and 31 (38.8%), respectively, had scheduling accommodations upon returning to work. Among residency programs with scheduling accommodations, 17 (35.4%) received a reduction in overnight shifts, 15 (31.3%) were exempt from overnight shifts, 12 (25.0%) were exempt from mandatory overtime/back-up call, and 3 (6.3%) had a standard reduction in total clinical shifts. However, 19 (39.6%) residency programs reported other accommodations, including increased flexibility based on resident preference, fewer consecutive shifts, and lighter rotations immediately following return. For attending physicians in programs that provided scheduling accommodations, 11 (35.5%) received fewer overnight shifts, 13 (41.9%) were exempt from overnight shifts, 15 (48.4%) were excused from mandatory overtime/back-up call, and 4 (12.9%) had clinical shift reductions after returning from leave. Like the residency programs, 11 (35.5%) attending groups reported other accommodations for attendings, including self-scheduling, increased schedule requests, and limited consecutive shifts.

**Table 5:** Return-to-work and fertility policies for residents and attendings in emergency medicine programs in the United States.

Policies	Residents	Attendings
Return-to-work accommodations		
Return-to-work accommodations provided, n (%)		
No	49 (48.0)	44 (53.7)
Yes	48 (47.1)	31 (38.8)
Unknown	5 (4.9)	7 (8.5)
Types of return-to-work accommodations (n = 48 for residents, n = 31 for attendings), n (%)		
Reduced numbers of overnights	17 (35.4)	11 (35.5)
No overnight shifts	15 (31.3)	13 (41.9)
No mandatory overtime/jeopardy	12 (25.0)	15 (48.4)
Fewer overall shifts	3 (6.3)	4 (12.9)
Other	19 (39.6)	11 (35.5)
Fertility		
Assisted reproductive technologies included in benefits package, n (%)		
No	19 (18.6)	17 (20.7)
Yes, full	32 (31.4)	29 (35.4)
Yes, partial	9 (8.8)	8 (9.8)
Unknown	42 (41.2)	28 (34.2)

### 3.5 Fertility Benefits

Assisted reproductive technologies, including egg harvesting, intrauterine insemination, and in vitro fertilization, were either fully or partially included in the benefits packages of 41 (40.2%) residency programs and 37 (45.1%) attending groups (Table 5). Most respondents either did not offer or were uncertain whether they offered comprehensive fertility benefits.

## 4 Limitations

We had a modest response rate (102/282 [36%] for residents, 83/278 [29%] for attendings), with respondents more likely to be university-based programs, which may introduce response bias. There was a relatively large proportion of “Unknown” responses in some questions, which may have overestimated or underestimated our findings, though again highlights the need for clear, accessible policies across all EM sites. Self-reported data also pose risks of recall or social desirability bias, and interpretations of survey questions may have varied due to multibarrel items or inadequate piloting and assessment of the survey prior to deployment. We did not verify the survey responses with official written policies. Therefore, the responses may reflect clinician perceptions or local practice patterns rather than formal policy. Additionally, as only EDs with residency programs were surveyed, results may not fully reflect practices in EDs without residency programs. We did not measure the time required to complete the survey and, therefore, could not characterize respondent burden.

## 5 Discussion

To our knowledge, this is the first national study to assess existing EM-specific parental policies for physician pregnancy, parental leave, and lactation. Based on our survey results, US EM programs’ parental policies for both residents and attendings do not well align with recent consensus recommendations developed by Lall et al,^26^ often failing to meet minimum standards. Despite the known risks to the birthing parent and fetus, most emergency physicians are not receiving a reduction in the total number of clinical shifts or in overnight shifts during the first trimester, and only 69% of residency programs and 76% of attending groups exempt physicians in their third trimester from overnight shifts. Although postnatal compensated leave was universally provided for birthing parents in this study, this benefit was less robust for nonbirthing parents and parents through adoption or surrogacy. Although dedicated lactation space was offered in most residency programs and attending groups, many were not equipped with a computer linked to the electronic health record. Additionally, most respondents did not eliminate or minimize single-coverage shifts during lactation. A minority of residency programs and attending groups offered return-to-work accommodations, such as a reduction in overnight shifts or an exemption from mandatory overtime. This not only negatively affects emergency physicians’ well-being but also poses a potential patient safety concern, given the increased risk of medical errors associated with physician sleep deprivation.^28^^,^^29^

The US is known to lag behind other developed nations in parental leave, which was again corroborated by the results of this survey, demonstrating inadequate parental leave. Paid parental leave has multiple benefits, including improved maternal physical and mental health, increased breastfeeding initiation and duration, reduced incidence of low birth weight, and reduced infant mortality.^7^^,^^30^ The benefits of paid parental leave for nonbirthing parents are important for the birthing parent, partner, and child, and include improved parent-child relationships, increased involvement in child care, better spousal relationships, more equitable division of family labor, and improved infant neurodevelopment.^30^^,^^31^ Despite consensus recommendations and evidence showing that the more parental leave is extended, the greater the benefits to parents and children, in the first 40 years of life, the group in our study with the most paid parental leave (attending birthing parents) received only 8 weeks.^32^

Nonbirthing parents, including fathers, nonbirthing mothers, and parents by adoption or surrogacy, were noted to have less paid leave than birthing parents. Further, approximately half to one-third of parents by adoption or surrogacy do not receive paid parental leave. This reflects an under-recognition of the role of nonbirthing parents in supporting the health and well-being of a newborn and ongoing systemic bias that negatively impacts families of both traditional and nontraditional structures. Including nonbirthing parents in paid parental leave policies helps normalize shared parental responsibilities, reduce gender-based resentment related to increased workload among peers to ensure adequate clinical coverage of an ED, and may mitigate bias in hiring junior faculty when parental leave expectations are equitable across genders.

Consistent with federal law, most programs had written lactation policies, dedicated private lactation spaces, and indefinite accommodations for breastfeeding parents. Although the elimination or reduction of single-coverage shifts while breastfeeding was not standard, most (89% of residency programs and 87% of attending groups) reported that physicians were able to safely take breaks from clinical care for the expression of breastmilk. Continuing clinical care while expressing milk, including the use of wearable pumps, should not be expected of lactating physicians, as produced milk volume and associated infant weight gain are influenced by maternal mental and emotional state while lactating.^33^^,^^34^ However, this option should be offered for those who prefer it, as it may help avoid post-shift work and documentation that contribute to the “breastfeeding penalty,” the systemic economic, professional, and career disadvantages experienced by parents who breastfeed after returning to work.^35^ The survey indicates that 66% of residency programs and 54% of attending groups had access to a desk/table with a telephone and computer connected to the electronic health record for clinical work, which leaves room for improvement. Fewer than half of the respondents had access to fertility coverage. Although some state and federal laws dictate minimum requirements for coverage of infertility treatment, employers and hospital groups can still choose to provide more comprehensive coverage than is mandated.

Prior to this study, it was known that there were inconsistencies and dissatisfaction with parental policies in EM.^36^ The significant variability in existing policies compared with the consensus recommendations for parental policies, such as schedule and shift modifications, leave duration, and lactation support, suggests that many programs currently have inadequate or unclear policies to support physicians during family building. Hospitals and EM physician groups should prioritize implementing and communicating the consensus recommendations for parental policies.^26^ Doing so may improve physician well-being, retention, and recruitment, particularly as the number of women in EM continues to grow. Reasonable accommodations for parents are often low-cost interventions that may reduce costly workforce attrition, increase institutional retention, and promote gender equity in academic advancement.

The strengths of this study include the use of a comprehensive survey that captured key aspects of parental support, including prenatal accommodations, lactation, and fertility benefits, across both residency programs and attending groups. Including academic and community sites enhances the relevance of findings and offers a foundation for future improvements in policy and practice.

The parental policies of US EM programs do not well align with recently published consensus recommendations for physician parents, providing an opportunity for improvement. Starting a family is an important milestone for many emergency physicians, and protecting physician parents and their children is a key strategy to accomplish this goal. These findings underscore the need for continued evaluation of parental policies in every ED and ongoing research to clarify how implementation and adherence to consensus recommendations affect parental and fetal outcomes, including psychosocial health, morbidity, and mortality.

## Author Contributions

LS and JHH conceived of the study, designed the survey, and supervised the data collection. JHH and WM managed statistical analysis. SC, JD, MD, CF, KH, RK, KM, ANM, SM, JP, RS, RS, and MY assisted with the development of the standard operating procedures, data collection, interpretation of the results, and drafting a portion of the manuscript. MDL served as the subject matter expert. LS drafted portions of the manuscript and revised the entire manuscript, LS, MDL, and JHH contributed significantly to its revision.

## Funding and Support

By *JACEP Open* policy, all authors are required to disclose any and all commercial, financial, and other relationships in any way related to the subject of this article as per ICMJE conflict of interest guidelines (see www.icmje.org). This work was supported by the 10.13039/100006108National Center for Advancing Translational Sciences (UL1 TR000445 from NCATS/NIH). Dr. Han received support from the Geriatric Research, Education, and Clinical Center and the Veterans’ Wellbeing through Innovation, Systems Science and Experience in Learning Health Systems (VETWISE-LHS).

## Conflict of Interest

All authors have affirmed they have no conflicts of interest to declare.

## References

[1] Stentz N.C., Griffith K.A., Perkins E., Jones R.D., Jagsi R. (2016). Fertility and childbearing among American female physicians. J Womens Health (Larchmt).

[2] Saak J.C., Mannix A., Stilley J., Sampson C. (2021). Diversity begets diversity: factors contributing to emergency medicine residency gender diversity. AEM Educ Train.

[3] 2024 Matriculating student questionnaire all schools summary report. https://www.aamc.org/data-reports/students-residents/report/matriculating-student-questionnaire-msq.

[4] Blair J.E., Mayer A.P., Caubet S.L., Norby S.M., O’Connor M.I., Hayes S.N. (2016). Pregnancy and parental leave during graduate medical education. Acad Med.

[5] Grunebaum A., Minkoff H., Blake D. (1987). Pregnancy among obstetricians: a comparison of births before, during, and after residency. Am J Obstet Gynecol.

[6] Guille C., Frank E., Zhao Z. (2017). Work-family conflict and the sex difference in depression among training physicians. JAMA Intern Med.

[7] Burtle A., Bezruchka S. (2016). Population health and paid parental leave: what the United States can learn from two decades of research. Healthcare (Basel).

[8] Cai C., Vandermeer B., Khurana R. (2019). The impact of occupational shift work and working hours during pregnancy on health outcomes: a systematic review and meta-analysis. Am J Obstet Gynecol.

[9] Mozurkewich E.L., Luke B., Avni M., Wolf F.M. (2000). Working conditions and adverse pregnancy outcome: a meta-analysis. Obstet Gynecol.

[10] Behbehani S., Tulandi T. (2015). Obstetrical complications in pregnant medical and surgical residents. J Obstet Gynaecol Can.

[11] MacVane C.Z., Puissant M., Fix M. (2022). Scheduling practices for pregnant emergency medicine residents. AEM Educ Train.

[12] Lawson C.C., Whelan E.A., Hibert E.N., Grajewski B., Spiegelman D., Rich-Edwards J.W. (2009). Occupational factors and risk of preterm birth in nurses. Am J Obstet Gynecol.

[13] Stocker L.J., Macklon N.S., Cheong Y.C., Bewley S.J. (2014). Influence of shift work on early reproductive outcomes: a systematic review and meta-analysis. Obstet Gynecol.

[14] Fernandez R.C., Marino J.L., Varcoe T.J. (2016). Fixed or rotating night shift work undertaken by women: implications for fertility and miscarriage. Semin Reprod Med.

[15] Zhang X., Lin D., Pforsich H., Lin V.W. (2020). Physician workforce in the United States of America: forecasting nationwide shortages. Hum Resour Health.

[16] Juengst S.B., Royston A., Huang I., Wright B. (2019). Family leave and return-to-work experiences of physician mothers. JAMA Netw Open.

[17] Davids J.S., Scully R.E., Melnitchouk N. (2017). Impact of procedural training on pregnancy outcomes and career satisfaction in female postgraduate medical trainees in the United States. J Am Coll Surg.

[18] Cooklin A.R., Rowe H.J., Fisher J.R.W. (2012). Paid parental leave supports breastfeeding and mother-infant relationship: a prospective investigation of maternal postpartum employment. Aust N Z J Public Health.

[19] Bering J., Pflibsen L., Eno C., Radhakrishnan P. (2018). Deferred personal life decisions of women physicians. J Womens Health (Larchmt).

[20] Clem K.J., Promes S.B., Glickman S.W. (2008). Factors enhancing career satisfaction among female emergency physicians. Ann Emerg Med.

[21] Rizvi R., Raymer L., Kunik M., Fisher J. (2012). Facets of career satisfaction for women physicians in the United States: a systematic review. Women Health.

[22] Lu D.W., Hartman N.D., Druck J., Mitzman J., Strout T.D. (2019). Why residents quit: national rates of and reasons for attrition among emergency medicine physicians in training. West J Emerg Med.

[23] Stack S.W., Eurich K.E., Kaplan E.A., Ball A.L., Mookherjee S., Best J.A. (2019). Parenthood during graduate medical education: a scoping review. Acad Med.

[24] Magudia K., Ng T.S.C., Bick A.G. (2020). Parenting while in training: a comprehensive needs assessment of residents and fellows. J Grad Med Educ.

[25] Lorch S.A., Peña M.M., Montoya-Williams D. (2024). Optimizing public policies for pregnancy and infant outcomes. JAMA Pediatr.

[26] Lall M.D., Jayaprakash N., Carrick A. (2024). Consensus-driven recommendations to support physician pregnancy, adoption, surrogacy, parental leave, and lactation in emergency medicine. Ann Emerg Med.

[27] Ray K., Burger C., Clark A.T. (2024). Development and results of a novel emergency medicine residency research immersion program. J Clin Transl Sci.

[28] Trockel M.T., Menon N.K., Rowe S.G. (2020). Assessment of physician sleep and wellness, burnout, and clinically significant medical errors. JAMA Netw Open.

[29] Horwitz A., Bar-Shachar Y., Ran-Peled D. (2023). Sleep of mothers, fathers, and infants: a longitudinal study from pregnancy through 12 months. Sleep.

[30] Dammann C.E.L., Montez K., Mathur M. (2024). Paid family and medical leave: policy statement. Pediatrics.

[31] Li X., Fu Y., Mao A. (2025). The power of presence: the impact of paternity leave on child health in China. Health Econ.

[32] Ruhm C.J. (2000). Parental leave and child health. J Health Econ.

[33] Levene I., Mohd Shukri N.H., O’Brien F., Quigley M.A., Fewtrell M. (2024). Relaxation therapy and human milk feeding outcomes: a systematic review and meta-analysis. JAMA Pediatr.

[34] Mohd Shukri N.H., Wells J., Eaton S. (2019). Randomized controlled trial investigating the effects of a breastfeeding relaxation intervention on maternal psychological state, breast milk outcomes, and infant behavior and growth. Am J Clin Nutr.

[35] Worthington R.O., Adams D.R., Fritz C.D.L., Tusken M., Volerman A. (2023). Supporting breastfeeding physicians across the training continuum: a call to action. Acad Med.

[36] MacVane C.Z., Fix M.L., Strout T.D., Zimmerman K.D., Bloch R.B., Hein C.L. (2017). Congratulations, you’re pregnant! Now about your shifts…: the state of maternity leave attitudes and culture in EM. West J Emerg Med.

