# Supplementary material for: Alignment of United States Emergency Medicine Programs’ Pregnancy, Parental Leave, and Lactation Policies With Recent Consensus Recommendations
Source: J Am Coll Emerg Physicians Open. 2026 Jun 13;7(4):100428. doi: 10.1016/j.acepjo.2026.100428 (PMC13279154; doi:10.1016/j.acepjo.2026.100428)
Supplement: Supplementary Tables 1-5 [file mmc1.pdf]

Table S1. Characteristics of Survey Respondents versus Non-Respondents

Program Characteristics	Residency Programs Respondents n=102	Residency Programs Non-Respondents n=180	Attending Groups Respondents n=83	Attending Groups Non-Respondents n=199
Type of Program				
University	50 (49.0%)	61 (33.9%)	41 (49.4%)	70 (35.2%)
Community with University Affiliation	35 (34.3%)	71 (39.4%)	35 (42.2%)	71 (35.7%)
Community without University Affiliation	17 (16.7%)	48 (26.7%)	7 (8.4%)	58 (29.2%)
Missing/Unknown	0 (0.0%)	0 (0.0%)	0 (0.0%)	0 (0.0%)

Table S2. Work modifications during the prenatal period for residents and attendings in emergency medicine programs in the US.

Question	Response	n	Residents	n	Attendings
Are pregnant people in the first trimester able to limit the number of overnight shifts they work?	No<br>Yes<br>Unknown	102	58 (56.9%)<br>40 (39.2%)<br>4 (3.9%)	83	49 (59.0%)<br>28 (33.7%)<br>6 (7.2%)
Is there a specific weekly limit to the number of overnight shifts?	Exempt<br>Specific #<br>Unknown	40	25 (62.5%)<br>3 (7.5%)<br>12 (30.0%)	28	16 (57.1%)<br>1 (3.6%)<br>11 (39.3%)
What is the weekly limit of overnight shifts? Median (IQR)	Shifts	3	2 (2, 2)	1	4 (4, 4)
For residents, are medical appointments excused absences?	No<br>Yes<br>Unknown	102	23 (22.6%)<br>75 (73.5%)<br>4 (3.9%)	-	-<br>-<br>-
Are pregnant persons (up to 20 weeks) granted shift coverage or able to activate sick call in the event of a miscarriage?	No<br>Yes<br>Unknown	102	8 (7.8%)<br>86 (84.3%)<br>8 (7.8%)	83	7 (8.4%)<br>73 (88.0%)<br>3 (3.6%)
Is there a limit to the number of days of sick call they are allowed to take?	No<br>Yes<br>Unknown	86	45 (52.3%)<br>31 (36.1%)<br>10 (11.6%)	73	52 (71.2%)<br>15 (20.6%)<br>6 (8.2%)
How many days are they allowed to take? Median (IQR)	Days<br>Unknown	31	10 (5, 20)<br>9 (29.0%)	15	10 (5, 45)<br>8 (53.3%)
Are pregnant persons with significant pregnancy-associated illness (e.g., hyperemesis gravidarum) supported with modifications to clinical work?	No<br>Yes<br>Unknown	102	10 (9.8%)<br>80 (78.4%)<br>12 (11.8%)	83	8 (9.6%)<br>60 (72.3%)<br>15 (18.1%)
When medically indicated, is prepartum leave financially compensated (e.g., when bedrest is medically indicated)?	No<br>Yes, fully paid<br>Yes, partially paid<br>Unknown	102	12 (11.8%)<br>53 (52.0%)<br>13 (12.8%)<br>24 (23.5%)	83	18 (21.7%)<br>29 (34.9%)<br>28 (33.7%)<br>8 (9.6%)
Are pregnant persons in the third trimester exempt from overnight shifts, if desired?	No<br>Yes<br>Unknown	102	23 (22.6%)<br>70 (68.6%)<br>9 (8.8%)	82	15 (18.3%)<br>62 (75.6%)<br>5 (6.1%)
At what gestational week does the overnight exemption start? Median (IQR)	Weeks<br>Unknown	70	28 (27, 29)<br>14 (20.0%)	62	28 (26.5, 29)<br>22 (35.5%)
Are pregnant persons in the third trimester limited to a maximum number of clinical hours per week?	No<br>Yes<br>Unknown	102	78 (76.5%)<br>17 (16.7%)<br>7 (6.9%)	82	72 (87.8%)<br>8 (9.8%)<br>2 (2.4%)
What is the maximum number of clinical hours per week? Median (IQR)	Hours<br>Unknown	17	60 (57, 60)<br>1 (5.9%)	8	60 (32, 60)<br>3 (37.5%)
Are pregnant persons in the third trimester exempt from any mandatory shift work above their contracted hours (e.g., mandatory overtime or jeopardy/back-up call)?	No<br>Yes<br>Unknown	102	44 (43.1%)<br>38 (37.3%)<br>20 (19.6%)	82	28 (34.2%)<br>45 (54.9%)<br>9 (11.0%)

At what gestational week does the mandatory shift work exemption start? Median (IQR)	Weeks<br>Unknown	38	29 (24, 30)<br>13 (34.2%)	45	28 (24, 29)<br>17 (40.0%)
For pregnant persons in the third trimester (29 weeks to delivery), is there a contingency plan to cover clinical shifts for pregnancy complications or onset of labor?	No<br>Yes<br>Unknown	102	10 (9.8%)<br>89 (87.3%)<br>3 (2.9%)	82	7 (8.5%)<br>73 (89.0%)<br>2 (2.4%)
For pregnant persons (all trimesters), how often are patients with possible tuberculosis, meningitis, or herpes zoster directed toward nonpregnant health care providers?	Never<br>Always<br>Sometimes<br>Unknown	102	6 (5.9%)<br>50 (49.0%)<br>19 (18.6%)<br>27 (26.5%)	82	9 (11.0%)<br>22 (26.8%)<br>28 (34.2%)<br>23 (28.1%)
If seeing patients with possible tuberculosis, meningitis, or herpes zoster, are pregnant persons (all trimesters) provided with exceptional infection control measures?	No<br>Yes<br>Unknown	52	4 (7.7%)<br>32 (61.5%)<br>16 (30.8%)	60	4 (6.7%)<br>45 (75.0%)<br>11 (18.3%)

Table S3. Postnatal parental leave policies for residents and attendings in emergency medicine programs in the US.

Question	Response	n	Residents	n	Attendings
Number of weeks of paid leave for the birthing parent, Median (IQR)	Weeks<br>Unknown	102	6 (6, 8)<br>5 (5.0%)	82	8 (6, 12)<br>6 (7.3%)
Beyond birthing parent medical leave, is any other form of parental leave compensated (i.e., birth mother or father, adoptive, surrogate parent)?	No<br>Yes<br>Unknown	102	12 (11.8%)<br>72 (70.6%)<br>18 (17.7%)	82	18 (22.0%)<br>56 (68.3%)<br>8 (9.8%)
Check all who have additional compensated leave after birth.					
Birthing parent, beyond medical leave addressed above	Yes	102	42 (41.2%)	82	37 (45.1%)
Non-birthing parent	Yes	102	69 (67.6%)	82	47 (57.3%)
Adoptive parent #1	Yes	102	53 (52.0%)	82	35 (42.7%)
Adoptive parent #2	Yes	102	49 (48.0%)	82	27 (33.7%)
Parent #1 by surrogacy	Yes	102	47 (46.1%)	82	28 (34.1%)
Parent #2 by surrogacy	Yes	102	44 (43.1%)	82	23 (28.0%)
In those who responded yes, # of weeks of leave:					
Birthing parent, beyond medical leave addressed above, Median (IQR)	Weeks<br>Unknown	42	6 (6, 8)<br>5 (11.9%)	37	6 (4, 12)<br>9 (24.3%)
Non-birthing parent, Median (IQR)	Weeks<br>Unknown	69	6 (4, 6)<br>3 (4.4%)	47	8 (6, 12)<br>3 (6.4%)
Adoptive parent #1, Median (IQR)	Weeks<br>Unknown	53	6 (6, 7)<br>4 (7.6%)	35	12 (6, 12)<br>2 (5.7%)
Adoptive parent #2, Median (IQR)	Weeks<br>Unknown	49	6 (6, 7)<br>3 (6.2%)	27	12 (6, 12)<br>2 (7.4%)
Parent #1 by surrogacy, Median (IQR)	Weeks<br>Unknown	47	6 (6, 8)<br>4 (8.5%)	28	8 (6, 12)<br>3 (10.7%)
Parent #2 by surrogacy, Median (IQR)	Weeks<br>Unknown	44	6 (6, 8)<br>3 (6.8%)	23	8 (6, 12)<br>3 (13.0%)
Are parents allowed to use their parental leave, excluding birthing parent medical leave, nonconsecutively for 1 year following the birth or adoption of a child?	No<br>Yes<br>Unknown	101	17 (16.8%)<br>61 (60.4%)<br>23 (22.8%)	82	22 (26.8%)<br>47 (57.3%)<br>13 (15.9%)

Table S4. Lactation policies for residents and attendings in emergency medicine programs in the US.

Question	Response	n	Residents	n	Attendings
Does your employer have a written policy explaining the objectives and accommodations provided to support lactating parents?	No<br>Yes<br>Unknown	101	9 (8.9%)<br>79 (78.2%)<br>13 (12.9%)	82	16 (19.5%)<br>52 (63.4%)<br>14 (17.1%)
How long do the accommodations for lactating parents established by your institution last?	Limited<br>Indefinite<br>Unknown	79	6 (7.6%)<br>67 (84.8%)<br>6 (7.6%)	52	6 (11.5%)<br>36 (69.2%)<br>10 (19.2%)
If limited, how many months, median (IQR)	Months<br>Unknown	6	12 (6, 12)<br>0 (0.0%)	6	12 (12, 12)<br>0 (0.0%)
Is there a dedicated private lactation space in or close to the emergency department?	No<br>Yes<br>Unknown	102	1 (1.0%)<br>98 (96.1%)<br>3 (2.9%)	82	7 (8.5%)<br>73 (89.0%)<br>2 (2.4%)
Please provide any accommodations within the lactation space					
Sink	Yes	98	63 (64.3%)	73	42 (57.5%)
Refrigerator	Yes	98	88 (89.8%)	73	54 (74.0%)
Comfortable chair	Yes	98	96 (98.0%)	73	71 (97.3%)
Closet space to store pumping equipment	Yes	98	49 (50.0%)	73	37 (50.7%)
Is a hospital-grade pump provided?	No<br>Yes<br>Unknown	98	47 (48.0%)<br>35 (35.7%)<br>16 (16.3%)	73	35 (48.0%)<br>21 (28.8%)<br>17 (23.3%)
Does this space contain a desk/table with a telephone and computer connected to the electronic health records for clinical work?	No<br>Yes<br>Unknown	98	17 (17.4%)<br>65 (66.3%)<br>16 (16.3%)	73	26 (35.6%)<br>39 (53.4%)<br>8 (11.0%)
Are lactating physicians able to safely schedule breaks from clinical coverage for the expression of breast milk?	No<br>Yes<br>Unknown	102	6 (5.9%)<br>91 (89.2%)<br>5 (4.9%)	82	4 (4.9%)<br>71 (86.6%)<br>7 (8.5%)
Does your workplace eliminate single-coverage shifts for lactating physicians until cessation of lactation (up to 1 year)?	No<br>Yes<br>Unknown	102	37 (36.3%)<br>39 (38.2%)<br>26 (25.5%)	82	43 (52.4%)<br>27 (32.9%)<br>12 (14.6%)
Does your workplace minimize single-coverage shifts for lactating physicians until cessation of lactation (up to 1 year)?	No<br>Yes<br>Unknown	102	34 (33.3%)<br>43 (42.2%)<br>25 (24.5%)	82	33 (40.2%)<br>39 (47.6%)<br>10 (12.2%)
If your physician's work area or charting space is amenable to a privacy screen, is this offered for physicians who would like to pump in the charting area, so they do not have to leave the department?	No<br>Yes<br>Unknown	102	77 (75.5%)<br>14 (13.7%)<br>11 (10.8%)	82	66 (80.5%)<br>11 (13.4%)<br>5 (6.1%)

Table S5. Return to work and fertility policies for residents and attendings in emergency medicine programs in the US.

Question	Response	n	Residents	n	Attendings
Are there scheduling accommodations implemented upon return to work for the birthing parent? (e.g., overnight shifts, number or length of shifts)	No<br>Yes<br>Unknown	102	49 (48.0%)<br>48 (47.1%)<br>5 (4.9%)	82	44 (53.7%)<br>31 (37.8%)<br>7 (8.5%)
Reduced # of overnights	Yes	48	17 (35.4%)	31	11 (35.5%)
No overnight shifts	Yes	48	15 (31.3%)	31	13 (41.9%)
No mandatory overtime/jeopardy	Yes	48	12 (25.0%)	31	15 (48.4%)
Fewer overall shifts	Yes	48	3 (6.3%)	31	4 (12.9%)
Other	Yes	48	19 (39.6%)	31	11 (35.5%)
How many weeks are these accommodations provided for? Median (IQR)	Weeks<br>Unknown	48	4 (4, 8)<br>14 (29.2%)	31	12 (7, 12)<br>11 (35.5%)
Please specify when the accommodations start	Postpartum<br>Returning to work<br>Unknown	48	11 (22.9%)<br>20 (41.7%)<br>17 (35.4%)	31	3 (9.7%)<br>13 (41.9%)<br>15 (48.4%)
If compensated by productivity, is there a reduction in Relative Value Units (RVU) expectations for pumping parents?	No<br>Yes<br>Unknown	24*	17 (70.8%)<br>1 (4.2%)<br>6 (25.0%)	42*	30 (71.4%)<br>7 (16.7%)<br>5 (11.9%)
Is there any adjustment to bonus compensation for pumping parents?	No<br>Yes<br>Unknown	32*	26 (81.3%)<br>1 (3.1%)<br>5 (15.6%)	52*	38 (73.1%)<br>7 (13.5%)<br>7 (13.5%)
Are assisted reproductive technologies (such as egg harvesting for fertility preservation, IUI, or IVF) included in your workplace's benefits package?	No<br>Yes, full<br>Yes, partial<br>Unknown	102	19 (18.6%)<br>32 (31.4%)<br>9 (8.8%)<br>42 (41.2%)	82	17 (20.7%)<br>29 (35.4%)<br>8 (9.8%)<br>28 (34.2%)

*n are for programs that have relative value units (RVU) or bonuses.

IUI, intrauterine insemination; IVF, in vitro fertilization
